# Tunability of the Superconductivity of NbSe_2_ Films Grown by Two-Step Vapor Deposition

**DOI:** 10.3390/molecules28031059

**Published:** 2023-01-20

**Authors:** Huihui Lin, Meijuan Chang, Xingjie Fu, Pengfei Li, Maoxin Chen, Luyan Wu, Fangqi Yang, Quan Zhang

**Affiliations:** 1 Department of Chemistry, National University of Singapore, Singapore 117549, Singapore; 2 Institute for Functional Intelligent Materials, Department of Materials Science and Engineering, National University of Singapore, Singapore 117575, Singapore; 3 Key Laboratory for Organic Electronics and Information Displays, Jiangsu Key Laboratory for Biosensors, Institute of Advanced Materials (IAM), Jiangsu National Synergetic Innovation Center for Advanced Materials (SICAM), Nanjing University of Posts and Telecommunications, Nanjing 210023, China; 4 College of Chemistry and Chemical Engineering, Hubei Normal University, Huangshi 435002, China

**Keywords:** NbSe_2_, superconductor, transition-metal dichalcogenides (TMDCs), two-step vapor deposition

## Abstract

Layered metallic transition-metal dichalcogenides (TMDCs) are ideal platforms for exploring their fascinating electronic properties at two-dimensional limits, such as their charge density wave (CDW) and superconductivity. Therefore, developing ways to improve the crystallization quality of TMDCs is urgently needed. Here we report superconductively tunable NbSe_2_ grown by a two-step vapor deposition method. By optimizing the sputtering conditions, superconducting NbSe_2_ films were prepared from highly crystalline Nb films. The bilayer NbSe_2_ films showed a superconducting transition temperature that was up to 3.1 K. Similar to the salt-assisted chemical vapor deposition (CVD) method, superconducting monolayer NbSe_2_ crystals were also grown from a selenide precursor, and the growth strategy is suitable for many other TMDCs. Our growth method not only provides a way to improve the crystalline quality of TMDC films, but also gives new insight into the growth of monolayer TMDCs. It holds promise for exploring two-dimensional TMDCs in fundamental research and device applications.

## 1. Introduction

Beyond graphene, two-dimensional (2D) TMDCs show excellent electrical, mechanical, thermal, and optical properties, which have great potential for applications in low-power, high-performance, and flexible electrical devices in the future [1,2,3,4]. Among them, metallic TMDCs are ideal platforms for exploring the effects of dimensionality on their fascinating electronic properties such as their charge density wave (CDW) [5,6,7,8,9,10] and superconductivity [11,12,13,14,15,16]. As a typical metallic TMDC, 2H NbSe_2_ is a superconducting material that possesses a distinct layered structure, composed of Nb atoms sandwiched between two layers of hexagonally close-packed Se atoms [17,18,19]. The 2D superconducting NbSe_2_ exhibits significantly different properties from its bulk as the layers are reduced [6,13,14]. For example, the charge density wave order was enhanced [6,13], the CDW order and superconductivity can coexist, and the Ising pairing was protected by spin-momentum locking in superconductivity [14,20].

As we know, defects in an ultrathin superconductor are a critical factor to determining its intrinsic 2D superconductivity [21,22,23,24]. In turn, the superconducting transition critical temperature (*T*_c_, the temperature where the sheet resistance drops to 10% of its normal state) is an important parameter to evaluate the crystallization quality of a 2D superconductor. Many methods have been developed to improve the crystalline quality of superconducting few-layer NbSe_2_, for example: (a) exfoliation from bulk single-crystal NbSe_2_ by the electrochemical exfoliation method [25,26,27,28,29]; (b) growth by salt-assisted CVD [22,30,31]; (c) growth by molecular beam epitaxy (MBE) under ultra-high vacuum [20,32,33]; and (d) growth of a wafer-scale NbSe_2_ film in oxygen-free conditions by a two-step vapor deposition method [23]. However, most of the above preparations of NbSe_2_ have either a lot of point defects or a small grain size, which reduces its environmental stability or *T*_c_. Therefore, it is still challenging to develop a reliable strategy to grow 2D NbSe_2_ with a large area, high crystalline quality, and high repeatability.

In this work, a superconductively tunable NbSe_2_ was grown by a two-step vapor deposition method (including physical vapor deposition (PVD) and chemical vapor deposition). The crystalline quality of Nb films was improved by optimizing the sputtering temperature and pressure. After the selenylation of the Nb films, the *T*_c_ of the final bilayer NbSe_2_ was ~3.1 K, which was 0.3 K higher than that of our reported grown NbSe_2_ films [23]. Significantly, monolayer single-crystal NbSe_2_ with a large lateral size of ~50 μm was grown, which was grown by the two-step vapor deposition method the first time. The monolayer NbSe_2_ showed a *T*_c_ of ~2.1 K, which increased by more than ~1.0 K compared with the previous CVD-grown single-crystal NbSe_2_. The developed two-step vapor deposition method to grow superconducting 2D NbSe_2_ is of great importance in both fundamental and technological fields.

## 2. Results and Discussion

**Figure 1:**
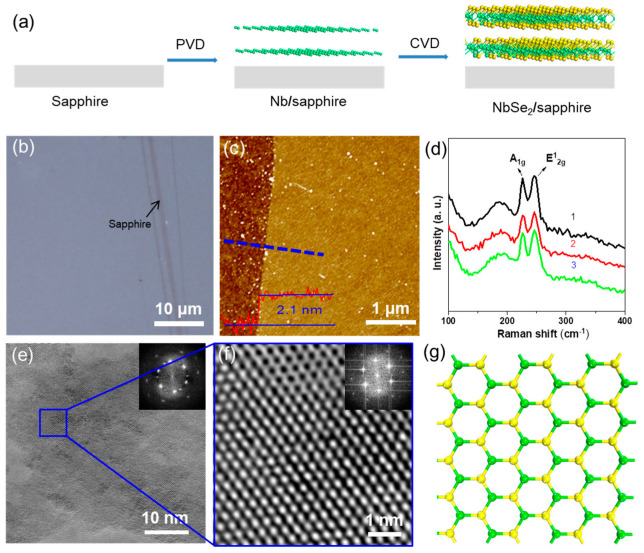
(**a**) A schematic illustration of the two-step vapor deposition method to grow 2D NbSe_2_; (**b**) optical microscopic image; (**c**) AFM image and the corresponding height profile; (**d**) Raman spectra (3 different sites of the as-grown NbSe_2_ were tested and represented as black, red and green lines, respectively) of as-grown NbSe_2_ film, the blue dotted line and red line are the measure site and corresponding height profiles, respectively; (**e**) a low-magnification TEM image showing the polycrystalline structure of NbSe_2_ film; and (**f**) the atomic structure image of the area marked out by a blue square in (**e**) shows a perfect single-crystalline structure (as confirmed by the inset fast Fourier transform pattern); (**g**) atomic model of NbSe_2_ (top view). The green sphere represents the Nb atom and the yellow sphere represents the Se atom.

A schematic of our synthesis method is illustrated in Figure 1a. The NbSe_2_ was successfully grown by the selenylation of Nb films. Figure 1b shows the optical images of the grown NbSe_2_ films on sapphire with excellent optical uniformity over the entire film. Figure 1c shows an atomic force microscope (AFM) image of NbSe_2_ films with a thickness of ~ 2.1 nm (corresponding to two layers), which is consistent with the reported works [22,34]. The as-prepared NbSe_2_ films showed two characteristic Raman peaks located at ~226 cm^−1^ and ~247 cm^−1^ (Figure 1d), which were assigned to A_1g_ (corresponding to an out-of-plane mode) and E^1^_2g_ (corresponding to an in-plane mode) modes respectively. The peak spacing between A_1g_ and E^1^_2g_ was ~21 cm^−1^, which is consistent with two layers of NbSe_2_ [6,8]. The energy dispersive spectroscopy (EDS) results of NbSe_2_ showed an atomic ratio of Nb: Se of approximately 2: 1 (Supplementary Figure S1), which is consistent with our previous work [23]. For further atomic structure characterization, the as-grown NbSe_2_ films were transferred onto a copper grid (Figure 1e,f). The atomic structure of NbSe_2_ (top view) is shown in Figure 1g. The low-resolution transmission electron microscope (TEM) image in Figure 1e shows a continuous polycrystalline film, as proved by the inset fast Fourier transform pattern. The zoomed-in image of a blue square in Figure 1e shows a typical high-resolution TEM image inside an individual grain, demonstrating a nearly perfect Nb atomic lattice across a 6 nm × 6 nm region (Figure 1f; only heavy atoms of Nb can be seen).

### 2.1. Optimization of the Crystalline Quality of Nb Films

**Figure 2:**
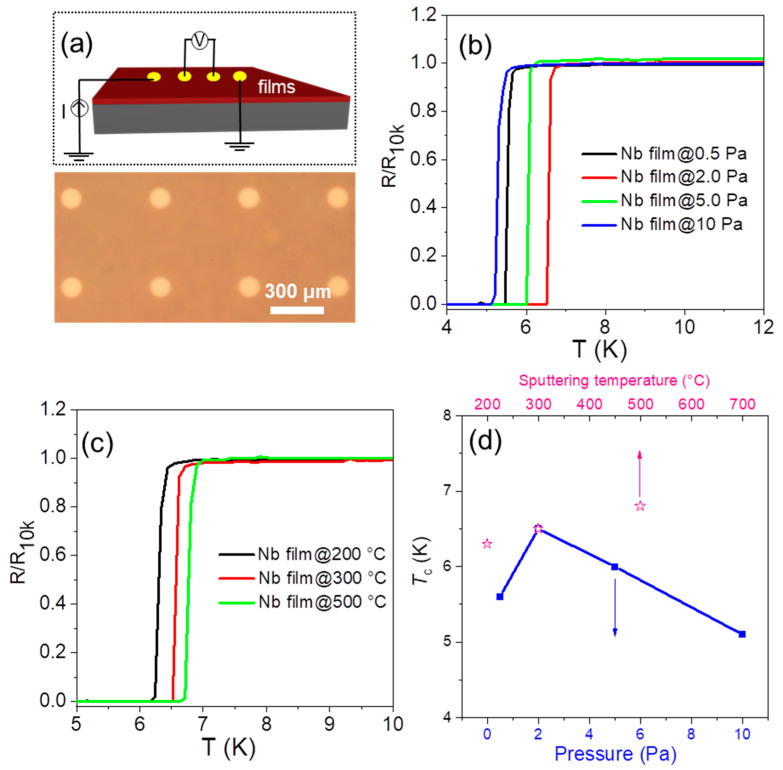
(**a**) A schematic illustration (top) and an optical image (bottom) of the four-probe contact configuration used in the transport experiments; superconductivity of Nb films at (**b**) different sputtering pressures (sputtering temperature: 300 °C) and (**c**) different sputtering temperatures (sputtering pressure: 2 Pa); (**d**) the *T*_c_ of NbSe_2_ films at different sputtering pressures (sputtering temperature: 300 °C) and temperatures (sputtering pressure: 2 Pa).

For relatively low-melting-point metals, such as Cu, its single-crystal films can be prepared by annealing sputtered Cu films at a temperature close to its melting point (~1085 °C). As for Nb metal with a high melting point, it is difficult to perform the annealing process near its melting point temperature (~2477 °C). In order to improve the crystalline quality of Nb films, the sputtering conditions were optimized (Supplementary Figures S2 and S3). As-sputtered polycrystalline Nb films below 10 nm showed semimetal behavior (Supplementary Figure S4), and the *T*_c_ of Nb films with a thickness of 28 nm was used to judge its crystalline quality (Supplementary Figure S3a). Figure 2a shows a schematic illustration (top) and an optical image (bottom) of the four-probe contact configuration used in the transport experiments. When the sputtering pressure increased from 2 Pa to 10 Pa, the sputtering rate increased correspondingly. At a higher sputtering rate, more atomic vacancies occurred in Nb films, leading to a higher resistance (Supplementary Figure S3b). As shown in Figure 2b, the Nb film sputtered at 2 Pa had the highest *T*_c_ of ~6.5 K (sputtering temperature is 300 °C). When the sputtering pressure decreased to ~0.5 Pa, the sputtering rate was unstable, so the Nb film with a lower *T*_c_ of ~5.5 K was sputtered. Usually, the sputtering rate will increase as the sputtering pressure increases. However, if the sputtering rate is too fast, then the sputtered particles are not easy to fuse on the substrate. If the pressure is too low, then the gas flow will be unstable and it is difficult to control the sputtering process, thus affecting the crystallization quality of the Nb film. Through our experiments, we found the sputtering rate at 2 Pa to be relatively low and stable, thus the final film had a high quality. At the optimized sputtering pressure of 2 Pa, we further studied the influence of the substrate temperature. The resistance decreased from 4.72 Ω to 3.71 Ω as the sputtering temperature increased from 200 °C to 500 °C (Supplementary Figure S5b). As shown in Figure 2c and Supplementary Figure S5a, when the sputtering temperature increased from 200 °C to 500 °C, the *T*_c_ of as-sputtered Nb films increased from 6.3 K to 6.7 K. Increasing the temperature of the substrate will provide additional energy for the merging of sputtering particles, thereby increasing the grain size of the Nb films. The Nb film with the highest crystalline quality was sputtered at the pressure and temperature of 2 Pa and 500 °C, respectively (referred as Nb film@500 °C), as shown in Figure 2d.

### 2.2. Tunability of Superconductivity in Bilayer NbSe_2_ Films

**Figure 3:**
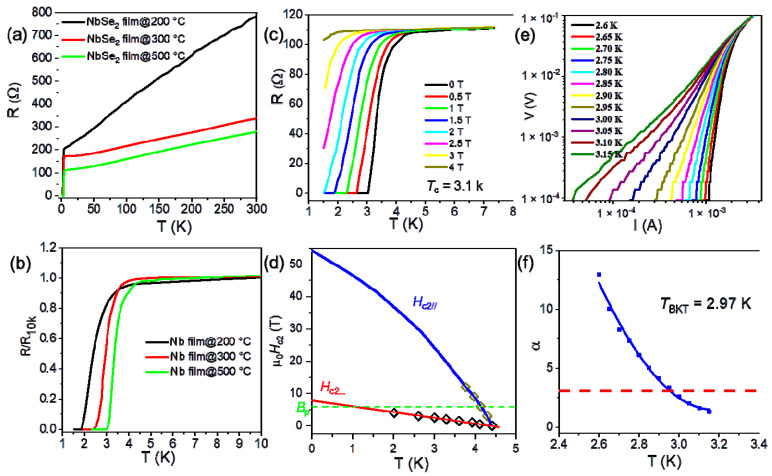
(**a**,**b**) Superconductivity of the NbSe_2_ films at the different sputtering temperatures; (**c**) the superconducting resistance transition of the NbSe_2_ film under an out-of-plane magnetic field; (**d**) temperature-dependence of the upper critical field *H*_c2_ under out-of-plane (red line) and in-plane (blue line) magnetic fields, the dashed green line indicates the Pauli limit field *B*_p_ = 5.7 T; (**e**) voltage–current (V–I) characteristics at different temperatures on a logarithmic scale; and (**f**) the corresponding BKT transition.

The tunability of the superconductivity of 2D NbSe_2_ makes it an ideal platform for tuning the intrinsic properties of TMDCs at 2D limits. The electrical transport properties of the as-grown NbSe_2_ films were studied. The temperature-dependent resistance of as-grown 2D NbSe_2_ from different crystalline Nb films are shown in Figure 3a,b. All the samples exhibited a metallic behavior (dR/dT > 0) at a high temperature range; the green lines show the temperature-dependent resistance of a bilayer NbSe_2_ (grown from Nb film@500 °C) at zero magnetic field with a current of 1 µA. The resistance began to decrease at 4.4 K and dropped to zero at 3.0 K. When the sputtering temperature increased from 200 °C to 500 °C, the superconducting transition temperature of the as-grown NbSe_2_ films correspondingly increased from 2.1 K to 3.1 K. The tunability of the superconductivity of 2D NbSe_2_ through the crystalline quality of Nb films can be used to tune the physical properties of other TMDCs.

The superconducting resistance transition of the bilayer film under out-of-plane and in-plane magnetic fields is shown in Figure 3c,d and Figure S6. As described by the Ginzburg–Landau theory of 2D superconductors [35], the coherence length and effective thickness of our NbSe_2_ film can be fitted to be ~6.5 nm and ~3.2 nm, respectively (Supplementary Figure S6). The effective film thickness is smaller than the coherence length, which proves that the as-grown NbSe_2_ films are 2D superconductors. The critical field at 0 K in the parallel (referred to as *B*_c_//(0)) and perpendicular (referred to as *B*_c_⊥(0)) magnetic fields were fitted to be ~54.0 T and ~7.9 T (Figure 3d), respectively. For Bardeen–Cooper–Schrieffer (BCS) superconductors, the Pauli paramagnetic limit (*B*_p_) can be simply rewritten as *B*_p_ = 1.84 *T*_c_ [14,36,37]. The *B*_p_ is estimated to be 5.7 T (*T*_c_~3.1 K). Thus, *B*_c_//(0) is 9.5 times higher than that of *B*_p_, proving Ising superconductor behavior in the as-grown NbSe_2_ films. The existence of such strong anisotropy provided direct evidence of the 2D character of the superconductivity of the as-grown NbSe_2_. The voltage–current characteristics at selected temperatures followed the Berezinskii–Kosterlitz–Thouless (BKT) model [38], and *T*_BKT_ was fitted to be 2.97 K when α reached 3 (Figure 3e,f). This value was close to the *T*_c_, indicating the occurrence of a BKT transition in the as-grown NbSe_2_ film.

### 2.3. Extending the Two-Step Vapor Deposition Method to Grow Monolayer NbSe_2_

**Figure 4:**
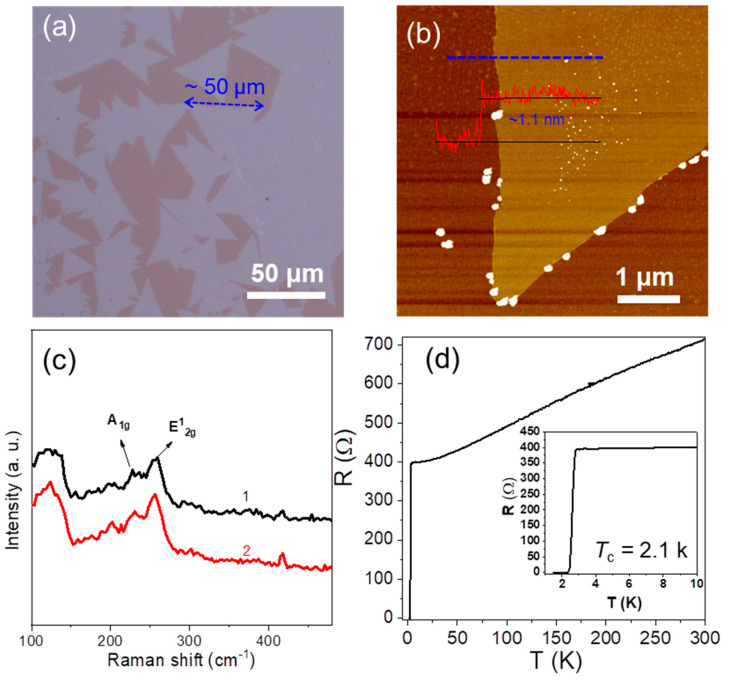
(**a**) Optical microscopic image; (**b**) AFM image and the corresponding height profile, the blue dotted line and red line are the measure site and corresponding height profiles, respectively; (**c**) Raman spectra (two different sites of the as-grown monolayer NbSe_2_ were tested and are represented as black and red lines, respectively); and (**d**) temperature-dependent resistance of monolayer NbSe_2_ crystal grown on sapphire.

To explore the potential of the two-step vapor deposition method, monolayer NbSe_2_ crystals were grown. As shown in Figure 4a, for the growth of monolayer NbSe_2_ crystals, a thin Nb film was firstly selenylated at 600 °C, which later was used as an active Nb source to grow NbSe_2_ crystals at 850 °C. Monolayer NbSe_2_ crystals with a large lateral size of ~50 μm can be grown. The thickness and Raman spectra corresponded to monolayer NbSe_2_ (Figure 4b,c). Due to at the limits of one layer, a weak intensity of Raman spectra was acquired at room temperature (Figure 4c). The TMDCs can decompose and re-combine at high temperatures, which can be used as a precursor to grow monolayer TMDCs. Thin NbSe_2_ was used as the Nb source to replace the precursor with one having a high melting point (e.g., Nb and Nb_2_O_5_), which ensured the self-limited growth of monolayer NbSe_2_ crystals. The temperature-dependent resistance of as-grown monolayer NbSe_2_ showed a *T*_c_ of 2.1 K (Figure 4d), which was higher than that of CVD-grown 2D NbSe_2_ crystals (Supplementary Table S1) [22]. The superconductivity of our grown monolayer NbSe_2_ was not vanished even without a protective layer, proving that the as-grown monolayer NbSe_2_ showed high stability. Monolayer MoSe_2_ crystals were also grown by the two-step vapor deposition method (Supplementary Figure S7). The growth of monolayer NbSe_2_ crystals by the two-step vapor deposition method has not been achieved in previous works, which shows great potential to grow other monolayer TMDCs.

## 3. Materials and Methods

### 3.1. Growth of NbSe_2_ Films and Monolayer NbSe_2_ Crystals

We used a two-step deposition method to grow NbSe_2_ films. The growth details were as follows: firstly, Nb (>99.9%) films were firstly sputtered on sapphire (heated at different temperatures); then, the Nb films were heated to 600 °C for 30 min in a two-zone furnace at zone II (600 °C). High-purity Se (>99.9%) was placed upstream at zone I (280 °C). A mixture gas of H_2_/Ar (1:10) was used as the carrier gas. The growth time for a homogenous film was 8 min. For monolayer NbSe_2_ crystals, a thin Nb film (<1 nm) was selenylated at 600 °C firstly, which was later used as an active Nb source to grow NbSe_2_ crystals at 850 °C. The as-grown samples were transferred by the traditional wet method to transmission electron microscopy (TEM) grids for further characterization.

### 3.2. Characterizations

TEM images were captured by FEI Titan 80-300, which was operated at 80 kV to minimize the knock-on damage. The samples were characterized by scanning electron microscopy (SEM) (FEI Verios 460 operated at 10 kV and 100 pA). Raman spectra were recorded using a Witec/alpha 300 R confocal microscope with a 532 nm laser at ambient conditions. AFM images were taken using the Bruker Dimension Icon in tapping mode. The superconducting and transport properties were analyzed in a 4He cryostat with a superconducting magnet (Oxford Teslatron 8 and 12 T), and electrical transport measurements were performed using a standard lock-in amplifier (Stanford SR830) with currents of 1~10 μA. Four-probe contacts were used by an e-beam-evaporated 80 nm Cr/Au array (the dots of array with 500 μm spacing).

## 4. Conclusions

In this study, the growth of high-quality superconducting 2D NbSe_2_ films was achieved. The crystalline quality of Nb films was improved by optimized the sputtering conditions. The highest *T*_c_ of ~3.1 K was achieved in the bilayer NbSe_2_, which was 0.3 K higher than our previously grown NbSe_2_ films. Superconducting monolayer NbSe_2_ crystals were grown from selenylated Nb films. Similar to salt-assisted CVD, which reduces the high melting points of the precursor by adding salt (e.g., NaCl), here we used thin NbSe_2_ as an Nb source to replace the precursor with one having a high melting point, and no foreign elements were introduced. This new growth technology will enrich the growth of other monolayer TMDCs, and particularly meet the challenge for the growth of TMDCs, whose metal/metallic oxides show a high melting point. Our method offers great versatility and controllability for the growth of 2D TMDCs (including 2D films, and even monolayer crystals) with improved crystalline quality, which paves the way for its fundamental research and further application in integrated devices.

## Data Availability

Not applicable.

## Supplementary Materials

The following supporting information can be downloaded at: https://www.mdpi.com/article/10.3390/molecules28031059/s1, Figure S1: Elemental analysis of as-grown NbSe_2_ film; Figure S2: AFM characterization of as-sputtered Nb film; Figure S3: AFM characterization of a 28 nm Nb film and transport measurement of as-sputtered Nb films at the different sputtering pressure; Figure S4: Transport property of a 5.5 nm Nb; Figure S5: Transport measurement of as-sputtered Nb films at the different sputtering temperature; Figure S6: Superconducting resistivity transition of the NbSe_2_ film under an in-plane magnetic field; Figure S7: Monolayer MoSe_2_ crystals grown by Two-Step Vapor Deposition; Table S1: The *T*_c_ of monolayerNbSe_2_ by different preparation methods. Refs. [14,20,22,27,33] are mentioned in Supplementary Materials.

## Sample Availability

Samples of the compounds are available from the authors.
