# Supplementary material for: Tunability of the Superconductivity of NbSe2 Films Grown by Two-Step Vapor Deposition
Source: Molecules. 2023 Jan 20;28(3):1059. doi: 10.3390/molecules28031059 (PMC9921890; doi:10.3390/molecules28031059)
Supplement: Supplementary file 1 [file molecules-28-01059-s001.zip › molecules-2176786-supplementary.pdf]

Supplementary Materials for

"Tunability of the Superconductivity of $NbSe_2$ Films Grown by Two-Step Vapour Deposition"

Huihui Lin[1*], Meijuan Chang[1, 2], Xingjie Fu[1], Pengfei Li[1], Maoxin Chen[1], Luyan Wu[3], Fangqi Yang[3*] and Quan Zhang[4*]

[1] Department of Chemistry, National University of Singapore, Singapore 117549, Singapore

[2] Institute for Functional Intelligent Materials, Department of Materials Science and Engineering, National University of Singapore, Singapore 117575, Singapore

[3] Key Laboratory for Organic Electronics and Information Displays, Jiangsu Key Laboratory for Biosensors, Institute of Advanced Materials (IAM), Jiangsu National Synergetic Innovation Center for Advanced Materials (SICAM), Nanjing University of Posts and Telecommunications, Nanjing 210023, China

[4] College of Chemistry and Chemical Engineering, Hubei Normal University, Huangshi 435002, China

* Correspondence: chmhuih@nus.edu.sg (H.L.); yangfq@email.ncu.edu.cn (F.Y.); zhangquen15@gmail.com (Q.Z.)

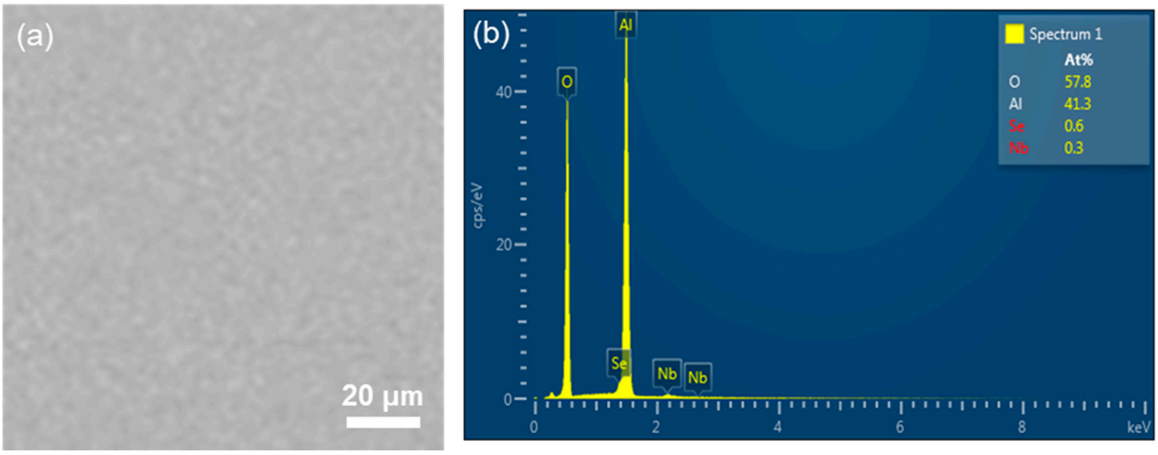

Supplementary Figure S1 Elemental analysis of as-grown $NbSe_2$ film. (a) SEM image and (b) EDS spectra of as-grown $NbSe_2$ film collected from a 100 μm × 100 μm region in Figure S1a.

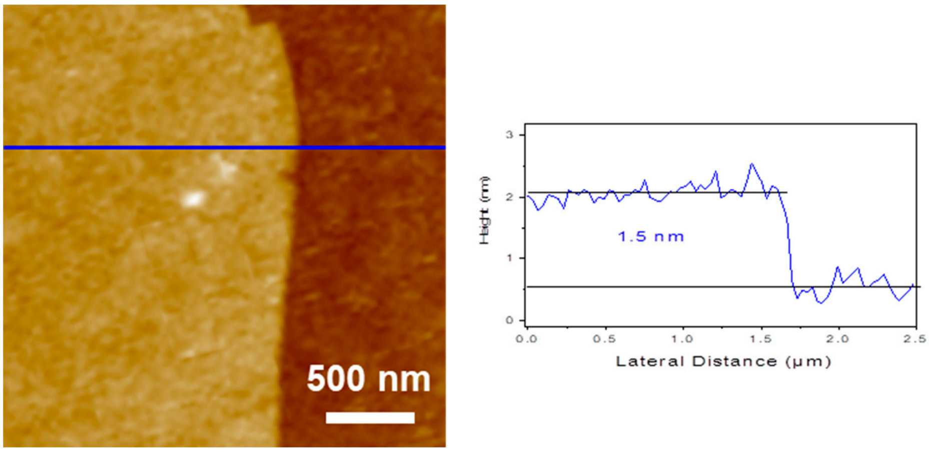

Supplementary Figure S2 AFM characterization of as-sputtered Nb film. AFM image and the corresponding height profile of a Nb film sputtered on sapphire.

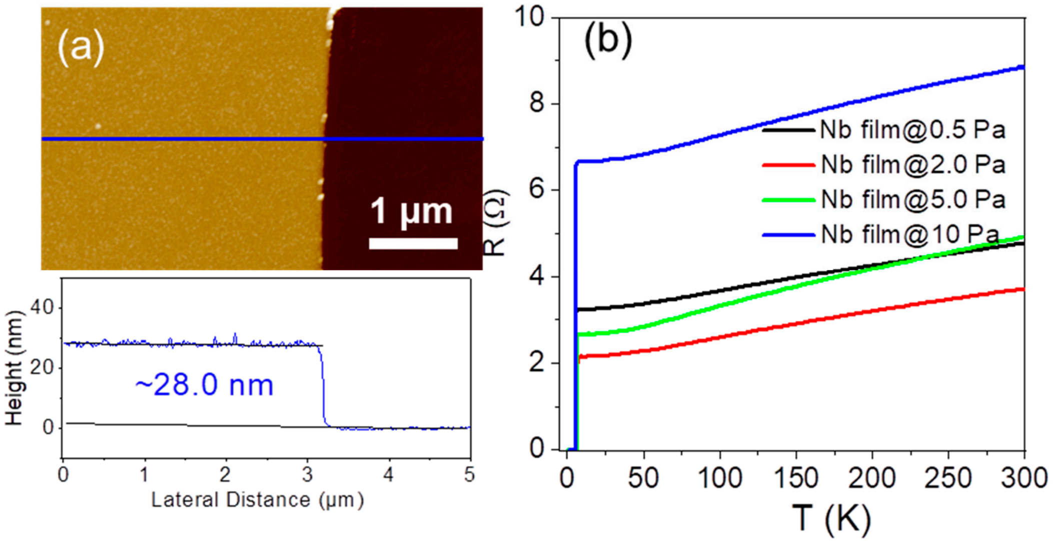

Supplementary Figure S3 AFM characterization of a 28 nm Nb film and transport measurement of as-sputtered Nb films at the different sputtering pressure. (a) AFM image and the corresponding height profile of an as-sputtered Nb film; (b) Superconductivity of as-sputtered Nb films at the different sputtering pressure. Note: The superconducting transition critical temperature (T_c): is the temperature where the sheet resistance drops to 10% of its normal state.

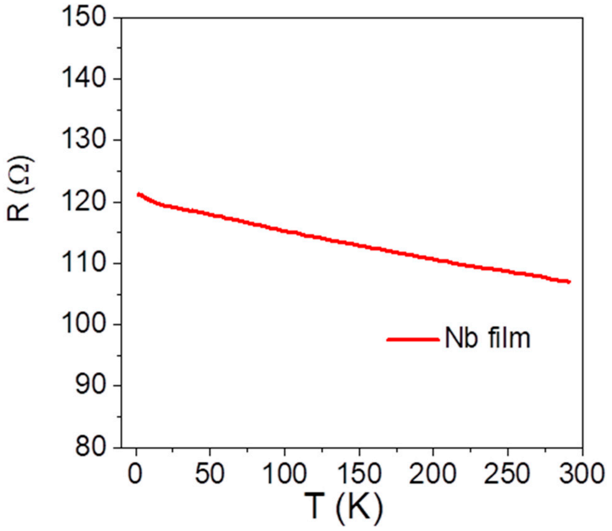

Supplementary Figure S4 Transport property of a 5.5 nm Nb. Temperature dependent resistance of a 5.5 nm Nb film, the film shows semimetal behavior (dR/dT < 0) and without the appearance of superconductivity.

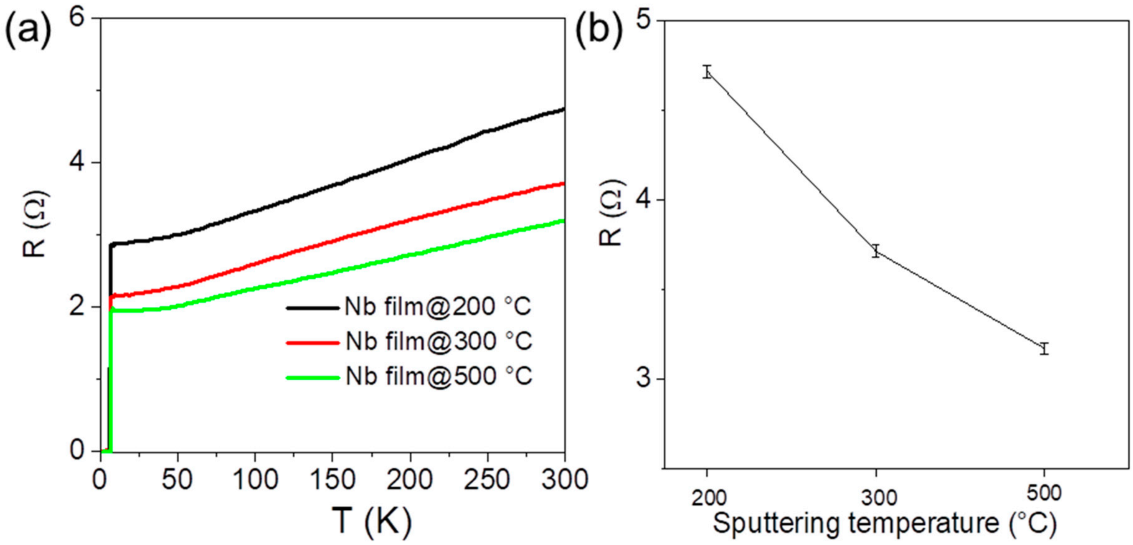

Supplementary Figure S5 Transport measurement of as-sputtered Nb films at the different sputtering temperature. (a) Superconductivity of as-sputtered Nb films at different sputtering temperature; (b) Resistance of as-sputtered Nb films at the different sputtering temperature (5 different sites are tested for every sample at room temperature).

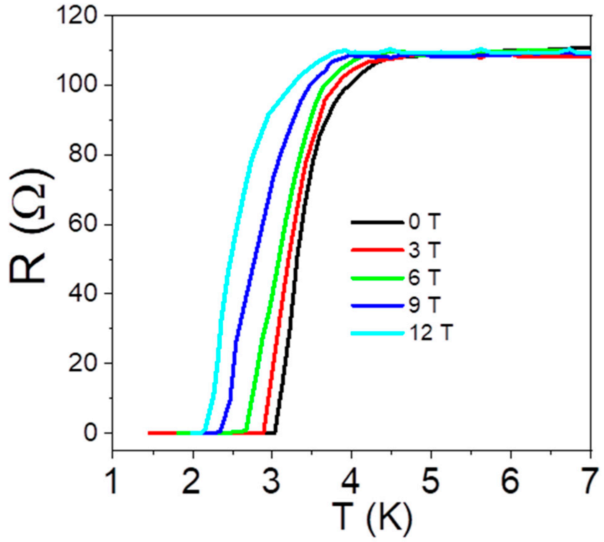

Supplementary Figure S6 Superconducting resistivity transition of the $NbSe_2$ film under an in-plane magnetic field.

As described by the Ginzburg-Landau (GL) theory of 2D superconductors, coherence length and effective thickness can be further derived from $H_{c2\parallel}$ and $H_{c2\perp}$ with the following relations:

$$H_{c2,\perp} = \frac{\Phi_0}{2\pi\xi_{GL}^2(0)}\left(1-\frac{T}{T_c}\right), \qquad H_{c2,\parallel} = \frac{\Phi_0\sqrt{12}}{2\pi\xi_{GL}^2(0)d_{SC}}\left(1-\frac{T}{T_c}\right)^{\frac{1}{2}}$$

where $\xi_{GL}(0)$ is the zero-temperature GL in-plane coherence length, Φ_0 is the magnetic flux quantum, and d_{SC} is the out-of-plane superconducting effective thickness. From the fitting, the coherent length is determined to be ~6.5 nm. An effective thickness of ~3.2 nm is obtained, which is smaller than $\xi_{GL}(0)$ and revealed 2D behavior of the superconductivity in the as-grown $NbSe_2$ film.

Supplementary Table S1 The T_c of monolayerNbSe$_2$ by different preparation methods.

Preparation method	Layers/ L	Protective layer	T_c/ K	Refs.
Tape exfoliation	1	Thin h-BN layer	~ 2.9	Nat. Phys. 2016, 12, 139. [14]
MBE	1	~ 10 nm amorphous Se layer	~ 0.50	Nat. Phys. 2016, 12, 92. [33]
MBE	1	20 nm amorphous Se layer	~ 0.65	Nano lett. 2017, 17, 6802. [20]
MBE	1	~ 20 nm amorphous Se layer	~ 1.0	Nature Physics, 2019, 15, 904. [1]
Electrochemical exfoliation	1	Thin h-BN layer	~3.2	Nat. Mater. 2021, 20, 181 [27]
Salt-assisted CVD	1	Graphene	~ 1.0	Nat. Commun. 2017, 8, 394 [22]
Two-step vapour deposition method	1	None	~ 2.0	This work

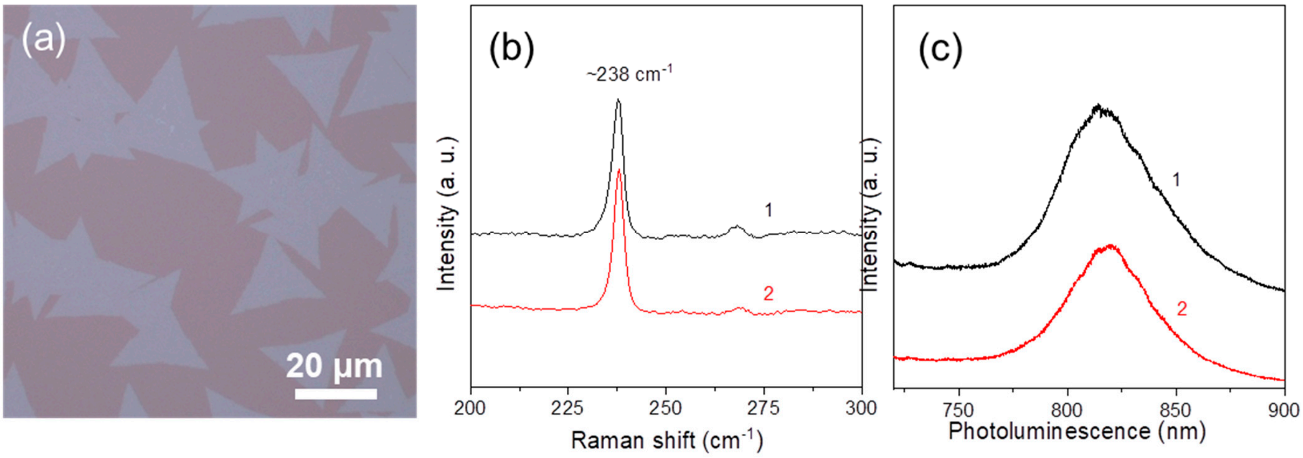

Supplementary Figure S7 Monolayer $MoSe_2$ crystals grown by Two-Step Vapor Deposition. (a) Optical microscopic image, (b) Raman spectra and (c) photoluminescence (PL) spectra of monolayer $MoSe_2$ crystals.

[1] Zhao, K. ; Lin, H. ; Xiao, X. ; Huang, W. ; Yao, W. and Yan, M. Disorder-induced multifractal superconductivity in monolayer niobium dichalcogenides. Nat. Phys. 2019,15, 904-910
